# Surface Damage Regeneration in Railway Wheels

**DOI:** 10.3390/ma19132930

**Published:** 2026-07-07

**Authors:** Krzysztof Labisz, Piotr Wilga, Jarosław Konieczny, Anna Włodarczyk-Fligier, Magdalena Polok-Rubiniec, Şaban Hakan Atapek, Janusz Ćwiek, Mateusz Winter

**Affiliations:** 1 Department of Railway Transport, Faculty of Transport and Aviation Engineering, Silesian University of Technology, Krasinskiego 8 Str., 40-019 Katowice, Poland; piotr.wilga@polsl.pl (P.W.); jaroslaw.konieczny@polsl.pl (J.K.); janusz.cwiek@polsl.pl (J.Ć.); mateusz.winter@polsl.pl (M.W.); 2 Faculty of Mechanical Engineering, Silesian University of Technology, Konarskiego 18A Str., 44-100 Gliwice, Poland; anna.wlodarczyk-fligier@polsl.pl (A.W.-F.); magdalena.polok-rubiniec@polsl.pl (M.P.-R.); 3 Laboratory of High Temperature Materials, Department of Metallurgical and Materials Engineering, Kocaeli University, 41001 İzmit, Türkiye; hatapek@kocaeli.edu.tr

**Keywords:** railway wheels, surface treatment, PTA, metal powders, plasma transferred arc

## Abstract

This study examines the applicability of Plasma Transferred Arc (PTA) surface treatment as an advanced technique for the refurbishment of railway wheel treads. Conventional wheel reprofiling, typically performed on semi-automatic lathes, requires the removal of a minimum of 6 mm of material from the running surface, which accelerates rim thinning and ultimately necessitates wheel replacement. Moreover, the reprofiled surfaces are not subjected to any subsequent treatment aimed at enhancing their durability. To overcome these limitations, PTA cladding was selected due to its ability to generate surface layers with superior mechanical and tribological properties. In contrast to widely used diode laser technologies, PTA enables the deposition of alloying materials in powder form, ensuring a stable, controllable, and efficient cladding process. The resulting microstructure consists of a heat-affected zone, a transition zone, and a re-melted zone, each exhibiting significantly increased hardness relative to the untreated base material. The process facilitates the incorporation of metallic particles into the surface layer, promoting the formation of a dense, wear-resistant coating. These materials possess huge potential utility regarding the wear resistance reaching even ca 10% of the base material wear in the case of 505 PTA and over 20% in the case of the 15 E material. The findings indicate that PTA surface treatment has substantial potential to extend the operational lifespan of railway wheels by providing a highly durable and mechanically robust surface, thereby reducing maintenance frequency and the associated costs.

## 1. Introduction

The degradation of railway rails and wheels results from numerous co-occurring and interacting mechanisms, the dominant one being rolling contact fatigue (RCF), which develops at the wheel–rail interface. Additionally, environmental and climatic factors, such as temperature fluctuations, humidity levels, and the presence of contaminants such as water, dust, and particulate matter, intensify wear and fatigue processes by modifying friction conditions, initiating corrosion, and destabilizing the lubrication film [1,2,3,4,5].

The wheel–rail interface is a complex tribological system in which mechanical, thermal, and chemical phenomena overlap. The interplay of these processes emphasizes the need for a comprehensive analysis of the microstructural and mechanical responses of the wheel material under conditions similar to those in service. Understanding these relationships is crucial for optimizing material selection, improving wheel design, and developing effective maintenance strategies to mitigate the negative effects of contact fatigue [6,7,8,9,10,11,12].

The chemical composition of steel, including alloying additives that influence hardness, impact strength, and fatigue resistance, determines the basic properties of the material. The microstructure—including grain size, phase distribution, and the presence of inclusions and precipitates—plays a key role in the initiation and propagation of fatigue cracks [13,14,15]. Heat treatment parameters used during wheel manufacturing are also crucial, as they modify the microstructure to shape mechanical properties such as hardness and residual stress distribution, thus influencing fatigue life. Together, these factors determine the material’s intrinsic ability to withstand cyclic loads without premature failure [16,17,18].

**Figure 1:**
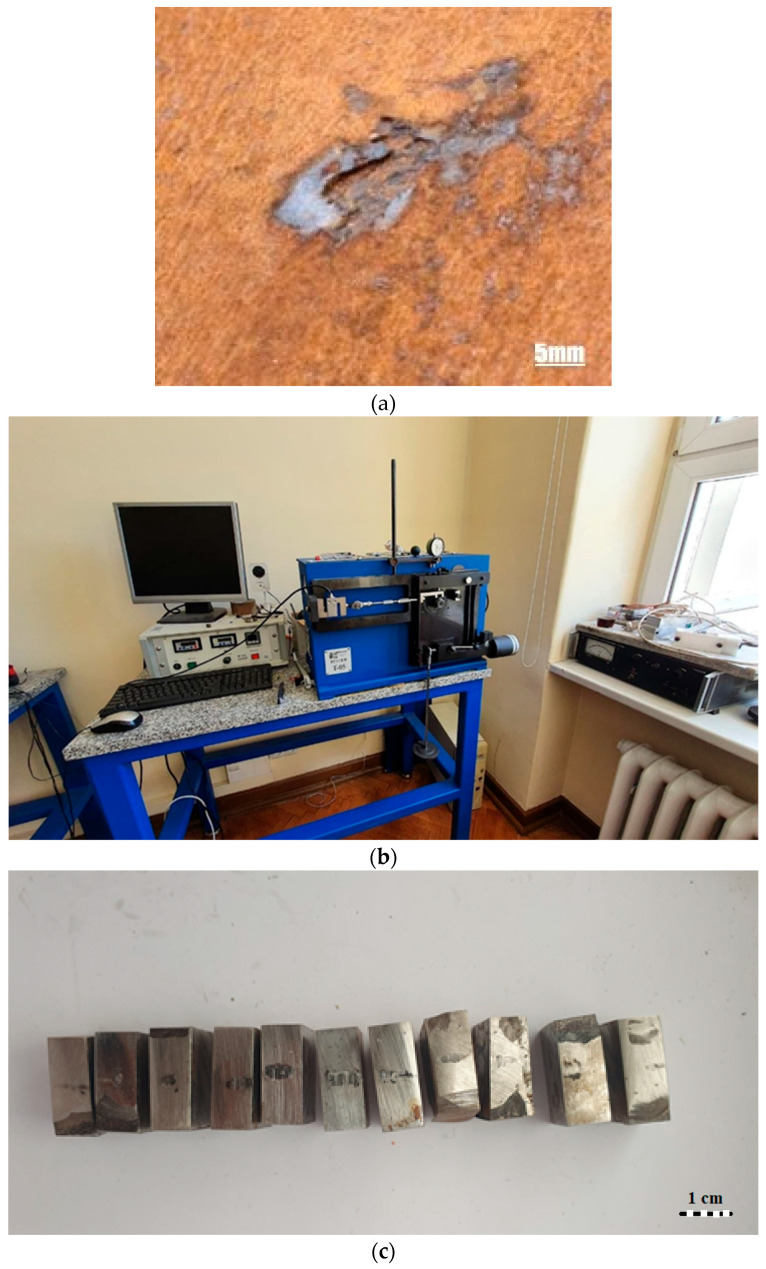
(**a**) Chipping—defects that occur on the running surface of the wheel (these are holes created by the detachment of the wheel’s native material); (**b**) testing machine used for investigations; and (**c**) samples using for testing.

Currently, carbon steel remains the primary material used for the production of railway wheels, valued for its favorable compromise between mechanical properties, availability, and cost. The manufacturing processes and quality requirements for railway wheels are detailed in the PN-EN 13262:2021-2 standard “Railway—Wheelsets and bogies—Wheels—Product requirements,” [19] which defines five steel grades approved for the production of monobloc railway wheels: ER6, ER7, ER8, ERS8, and ER9. The proper selection of steel grades is also crucial in the context of the environmental impact of rail transport, as processes such as the wear of wheels, rails, and braking components, as well as emissions associated with fuel combustion, contribute to soil and water degradation [20,21,22]. Among these grades, ER7 steel is widely used in industrial practice and frequently analyzed in scientific research due to its favorable set of mechanical properties and widespread use in railway networks [23,24,25]. Typical forms of damage observed during operation include surface wear, RCF cracks, chipping, surface layer detachment, and other fatigue defects. Examples of rolling surface damage are shown in Figure 1a, demonstrating the diversity and scale of wear phenomena. Figure 1b,c present the test rig and samples used in the analyses. The abrasion resistance tests were performed on the T-05 tester (Figure 1b) made by the company ITC.

Railway wheels are critical safety components that must meet strict engineering requirements related to strength, hardness, fatigue resistance, and dimensional stability during long-term operations. Due to high-contact stresses occurring at the wheel–rail interface, wheel materials must provide adequate resistance to wear, rolling contact fatigue (RCF), impact loading, and thermal loads generated during braking. According to the PN-EN 13262:2021-2 standard, railway wheels must also maintain a controlled chemical composition, microstructural homogeneity, and specific mechanical properties, including tensile strength, yield strength, and impact toughness. Additionally, the wheel profile and hardness distribution must ensure reliable vehicle guidance, minimize derailment risk, and reduce excessive wear of both wheels and rails. These requirements make the manufacturing and repair processes highly demanding, especially for wheels operating under heavy freight loads and intensive service conditions.

Recent advances in materials science and engineering have focused on elucidating the microstructural changes that occur during the service life of railway wheels. These efforts include the design of alloy compositions tailored to resist crack initiation, heat treatments to refine microstructures, and surface engineering techniques such as Plasma Transferred Arc (PTA) treatments to produce wear-resistant coatings. This knowledge base supports the development of engineering solutions that effectively mitigate rolling contact fatigue, ensuring the long-term operational reliability of railway infrastructure [26,27,28].

Research efforts aimed at characterizing microstructural changes, mechanical behavior, and the wear mechanisms of PTA-treated wheels will provide critical information necessary for the widespread adoption of these advanced surface engineering solutions in the railway industry. Continued research and development in this field will allow the railway sector to take advantage of these advances, ultimately contributing to safer, more reliable, and more cost-effective rail transport systems [19,20].

Concerning the limitations of existing railway wheel repair methods, current methods are primarily based on reprofiling by machining, which removes damaged material from the wheel tread and flange. Although this process restores the wheel’s geometry, it significantly reduces the wheel’s diameter and shortens the operational life of the wheelset. In cases of severe local defects, cracks, or excessive wear, wheels often need to be scrapped even when only a small region is damaged. Existing repair technologies such as welding and laser cladding also present several limitations, including residual stresses, heat-affected zones, microstructural changes, and the risk of crack initiation at the interface between the deposited material and the base metal. Additionally, maintaining uniform hardness and mechanical compatibility between the repaired layer and the parent material remains challenging. These limitations indicate the need for further research on advanced regeneration technologies capable of extending wheel service life while maintaining the strict safety and durability requirements of railway applications.

In parallel with microstructural characterization, the research evaluates the mechanical property enhancements afforded by the alloying additions. Hardness measurements, tensile testing, and fatigue resistance assessments are performed to quantify improvements in surface hardness, strength, and durability. The findings of this research contribute to a broader understanding of surface engineering through PTA treatment and underscore the potential of metal powder alloy additions as a versatile tool for tailoring surface properties [21,29]. The insights gained from this study also pave the way for future innovations in surface treatment technologies that aim to address the challenges posed by wear and fatigue in critical mechanical components such as railway wheels [22,30] and can also be investigated in combination with rail steel regarding microstructure changes and mutual abrasive wear [31]. Using a model that implements the optimization of multiple criteria with a set of proposed objective functions allows for the extension of the life span [32].

Generally, the research gap is primarily concerned with the lack of systematic studies on the abrasive wear of railway wheel flanges reconditioned using the PTA method, while most available studies focus on the RCF of the rolling surface. The influence of the chemical composition of the alloy powders used on the properties of the hard-facing layers in terms of abrasion resistance under the flange’s operating conditions also remains insufficiently understood. Furthermore, the literature lacks studies combining microstructural analysis with the assessment of mechanical and tribological properties for this specific wheel area. This paper fills this gap through targeted research on the properties and wear of PTA layers dedicated to wheel flange reconditioning. In this paper, the abrasive wear has been investigated to fill the research gaps, as a high number of old wheels are still available for use. The second reason was that the rail wheel flange—which is the subject of this study—is exposed to abrasive wear, not pressure during wheel–rail contact. Many railway wheels are withdrawn from service solely due to the excessive wear of the flanges.

This paper can also help to investigate the following issues: the general applicability of PTAs for rail wheel renovation, the influence of powders’ chemical composition on the suitability/hardness for wheel surfacing, and wear resistance.

## 2. Experimental Procedure

For this investigation, the most widely used railway grade steel, ER7, was used. The detailed chemical composition of this steel is provided in Table 1. The chemical compositions of the PTA powders are presented in Table 2. All the data presented in Table 1 and Table 2 were provided by the manufacturer. The producer was the Durmat company (Spring, TX, USA). Detailed specifications are presented below:

Firstly, 61 PTA is a material in the form of a metallic powder whose main characteristics are as follows:

- high resistance to abrasive wear;
- high corrosion resistance;
- acid resistance;
- resistant to abrasive processes;
- resistant to high temperatures. This material is more resistant to mechanical and mineral wear.

Secondly, 505 PTA is characterized by the following:

- high impact resistance;
- high abrasion resistance;
- good ductility. Moreover, 505 PTA is used;
- in mining;
- for brick and clay scraper blades;
- in agricultural machinery.

The advantages of 505 PTA include its ease of machining; this material can be machined by both turning and grinding processes.

Thirdly, 15 E’s coatings are very dense and corrosion-resistant. They are resistant to wear from abrasive grains, hard surfaces, particle erosion, and cavitation, at both low and high temperatures. 15 E is recommended for the most demanding service requirements when used on base materials with a relatively high coefficient of thermal expansion. The properties of coatings made from 15 E are as follows:

- resistant to abrasives and abrasive grains;
- resistant to hard surfaces;
- resistant to fretting;
- resistant to cavitation;
- resistant to particle erosion.

Typical applications for 15 E include the following:

- piston rings;
- cylinder liners;
- wear rings;
- water turbines;
- hydraulic pumps;
- dust collectors;
- exhaust valves;
- exhaust valve seats.

Finally, 36 C can be used in all applications requiring a high-quality, wear-resistant coating. Alloy 36 C is a material that, after being applied to the substrate material, must be melted or fused, resulting in a dense, pore-free coating. A coating made of 36 C exhibits the following properties:

- highly resistant to abrasion by abrasive grains;
- resistant to contact with hard surfaces;
- resistant to fretting;
- resistant to particle erosion.

The primary applications for 36 C include the following:

- piston rods;
- slurry pumps in the petroleum industry;
- capstans for pipe pulling;
- servo motor shafts.

Powder cladding is one of the most commonly used plasma cladding methods, and uses metallic and ceramic powders. Depending on the desired functional properties of the cladding process, powders vary in composition and diameter, in this case 2 mm. The melting efficiency depends on the chemical composition and ranges from 90 to 95%. The thickness of the coatings produced in a single process ranges from 0.25 to 0.7 mm. Due to the low heat input, this cladding technology enables the cladding of small components, even those with a thickness of 3–5 mm or a diameter of 20–50 mm. For the process, a shield gas argon was used with a flow rate of 13.0 L/min. The electrical current was 90 A with a voltage of 0.1 V.

Microstructural analysis was performed using a Carl Zeiss Suzhou Co. Stemi 508 doc body light microscope (Suzhou, China), serial number 3951004181, mounted on a stand k mat, serial number 43525-9020-000, with an Axiocam 105 color camera, serial number 3945011140. Surface observations were made at four magnifications: 50×, 100×, 200×, and 400×. Before testing, the sample was degreased and cleaned of any residual deposits caused by temperature and friction during the abrasive wear test. Numerous micrographs were also taken, showing the surface appearance of the samples immediately after the surfacing process and the abrasive wear test.

For detailed submicron-scale microstructure analysis, transmission electron microscopy (TEM, JEOL 3010UHR, Tokyo, Japan) was used. Both bright field imaging and electron diffraction (SAD) modes were used, providing insights into crystallographic data information. TEM enabled the direct observation of nanoscale features critical to understanding the alloy’s strengthening mechanisms, like the d-spacing value and phase analysis.

**Figure 2:**
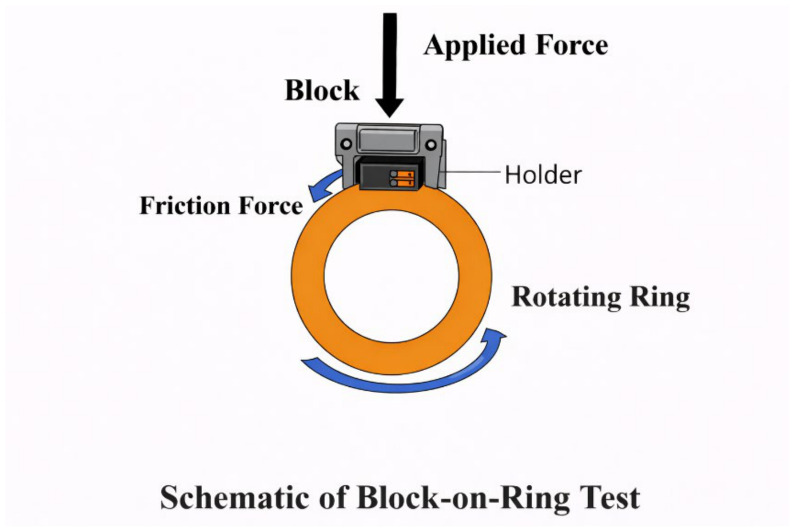
The Block-on-Ring test device used for investigation.

Abrasion resistance tests were performed using the Block-on-Ring method on a T-05 tester manufactured by ITC (Figure 2). A total of 17 cuboid samples were tested, with dimensions of 16 mm × 7 mm × 10 mm, made of five different materials. The following loads were used: 100 N 300 N, and 500 N, and the counter-sample rotational speeds were as follows: 100 rpm 200 rpm, and 300 rpm. The duration time was from 19 s to 179 s. The Block-on-Ring test parameters were selected to replicate the characteristic friction and abrasive wear conditions found in the wheel flange zone, where lower-pressure contacts but intense sliding interactions dominate. The loads and speeds used were selected to correspond to the actual ranges of contact stresses and relative sliding speeds observed in operations, particularly when negotiating track curves.

Surface hardness tests were performed on a Rockwell hardness tester, manufactured by KABID-PRESS in Warsaw, Poland, type KP15001, serial number 1237, manufactured in 1987. The hardness test was performed on the C scale. The samples were tested by applying a load of 150 kg through a sintered carbide cone-shaped indenter with an aperture angle of 120 degrees and a dwell time of 10 s. The measurements were repeated five times for each sample. The tests were carried out according to the PN-EN 6508-1 standard [33]. After measurement, the values were recalculated to the HV scale. The recalculated hardness values of 725 HV (0.82 GPa), 940 HV (1.23 GPa), and 1250 HV (2.04 GPa) were presented using the comparable GPa scale.

For the investigations, the following testing conditions were systematically applied to evaluate the material behavior and performance under various operational scenarios.

- Load levels of 450 MPa, 650 MPa, and 850 MPa;
- Rotational velocities of 0.183 m/s, 0.366 m/s, and 0.549 m/s.

## 3. Results and Discussion

**Figure 3:**
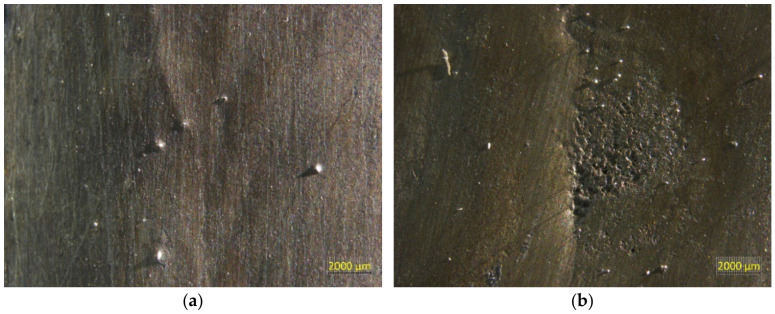
(**a**) Surface layer of the 61 PTA-treated material; (**b**) surface layer of the 36 C-treated material.

The Plasma Transferred Arc (PTA) method was used for surface alloying, utilizing metallic powders combined with alloy powders to create layers with improved wear resistance. The microstructural characteristics of the resulting layers are presented in Figure 3a,b. Clear differences are observed between surfaces alloyed with the NiBSiWC/36 C composite and surfaces modified solely by the PTA method. The layer treated solely with PTA exhibits a more uniform and regular morphology, devoid of visible surface defects. However, surfaces alloyed with NiBSiWC and 36 C materials exhibit distinct imperfections, such as gas bubbles and irregularities in the fusion zone. However, the microstructure reveals similarities to those presented in other works [34], where the transition zones were observed to be thin (approx. 9 µm to approx. 11 µm for different treatment methods like PPTAW), with a coarse-grain heat-affected zone (CGHAZ), made up of martensite laths, and peninsula-like macrosegregation observed for all. However, the PPTAW clad had cellular–dendritic growth solidification (CDGS) and a type-II boundary at the TZ, which is explained as resulting from the thermal cycles of this method; in this investigation, the microstructure does not reveal dendritic segregation, which is seen as positive due to the fact that the dendritic microstructure usually reveals lower mechanical properties.

**Figure 4:**
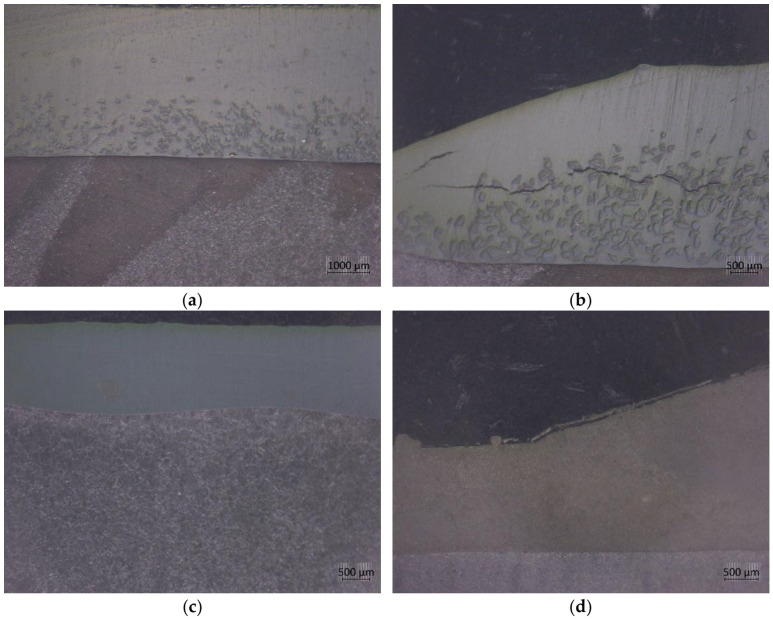
(**a**) Cross-section of the surface layer after feeding of the 36 C material, (**b**) 61 PTA material, (**c**) 15 A material, and (**d**) 505 PTA material.

Structural studies conducted at both macro- and microstructural scales enable comprehensive assessment of surface layers, including analysis of the shape and depth of the fusion zones. Examples of alloyed layers obtained through the surface modification process are shown in Figure 4a–d. Based on observations, it can be concluded that the type of alloying material used significantly affects both the penetration depth and the surface roughness after machining. It is worth emphasizing that the roughness of a railway wheel’s rolling surface is not a fixed value, but a set of requirements resulting from standards (profiling, shape) and operating instructions (permissible surface defects, Ra/Rz parameters). These parameters are intended to ensure the safe and stable rolling of the wheel on the rail, minimizing wear and noise emissions. PTA-derived layers are characterized by high abrasive wear resistance, which is associated with the presence of hard phases and a favorable microstructure. The comparison confirms the consistency of the observed trends, pointing to the significant role of the chemical composition of the additive material and its microstructure in shaping tribological properties. A supplemental literature analysis allows the obtained results to be better aligned with the current state of knowledge and their scientific contributions to be more clearly defined [34].

A distinctive zone-like structure was observed in the cross-section of the treated surfaces, characterized by the presence of a remelting zone comprising the molten fed powder material, an intermediate transition zone, and the underlying substrate material. In certain cases, a heat-affected zone (HAZ) was also identified, particularly in samples treated with the 15 E alloy, where noticeable growth was observed due to thermal exposure during processing. The measured thicknesses of the surface layers varied depending on the alloying material used, with the following values recorded: 15 E—approximately 2500 µm, 61 PTA—1500 µm, 36 C—4000 µm, and 505 PTA—3000 µm. It is important to emphasize that only materials 15 E and 36 C produced surface layers with relatively uniform thickness and consistent quality, exhibiting the absence of cracks, porosity, or unevenness, as documented in Figure 4. These findings highlight that the choice of alloying material and the processing parameters critically influence not only the microstructural characteristics, but also the integrity and homogeneity of the remelted layers. The uniformity and defect-free nature of the layers obtained with alloys of 15 E and 36 C suggest their greater suitability for applications that require enhanced wear resistance and structural reliability.

**Figure 5:**
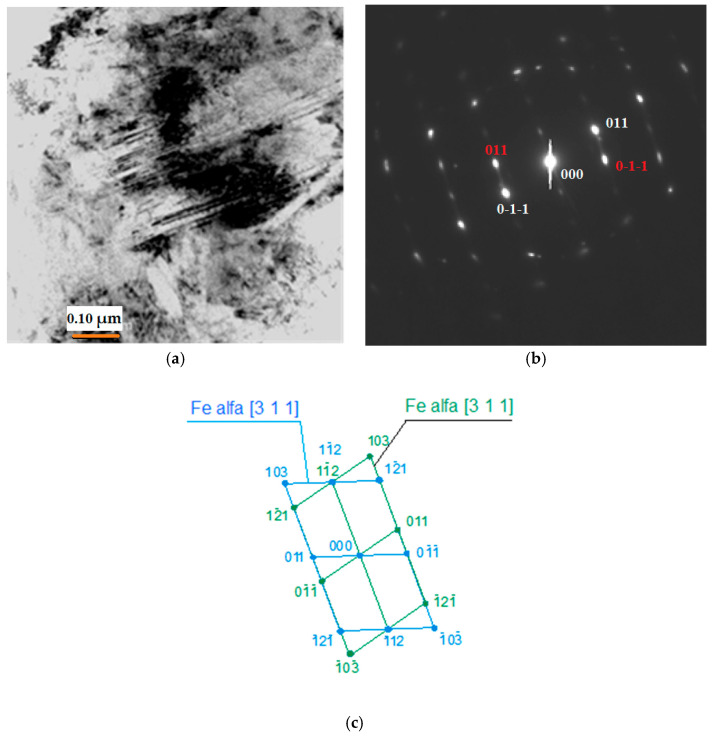
The structure of a thin foil of the tested steel after heat treatment of the surface, (**a**) bright field image, (**b**) diffraction pattern from the area shown in figure (**a**), and (**c**) solution of the diffraction pattern from figure (**b**).

During thermal spraying, the rim temperature remains low enough that the steel’s structure is virtually unchanged. The wheel’s massiveness effectively dissipates heat, reducing the possibility of local overheating. As a result, any tempering is minimal and superficial and does not affect the rim’s performance. There is also no hardening or phase transformation leading to the formation of ferrite (Figure 5). The entire process leaves the steel in a state close to its original condition.

**Figure 6:**
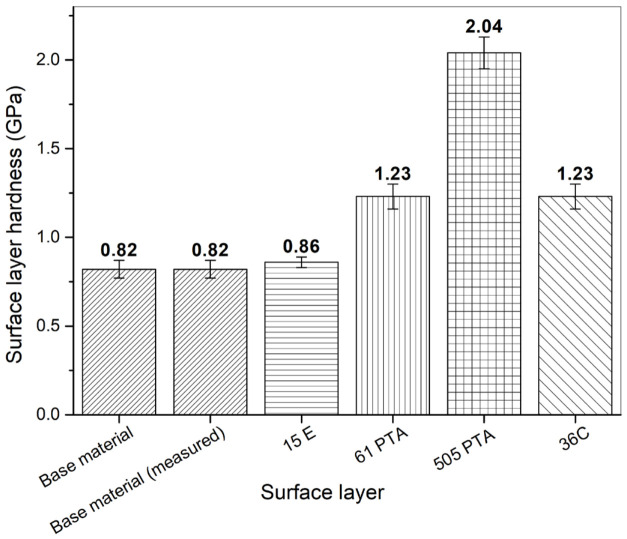
Surface hardness of the treated steel surface layer.

Figure 6 presents the measured hardness values of the surface layers obtained via the alloying process. It is obvious from the data that the hardness of the remelted zones is significantly higher than that of the base (parental) material, which exhibits a hardness of approximately 725 HV. Among the tested materials, the highest hardness value was recorded for the 505 PTA-treated layer. However, it is important to note that such elevated hardness levels, as observed in the 505 PTA layer, reaching even 248% compared to the base material, are generally not suitable for the repair of railway wheels. This is mainly because the hardness of the wheel surface should not exceed that of the corresponding rails. The excessive hardness of the wheel can lead to the accelerated wear of the rail, resulting in increased maintenance costs and potential operational inefficiencies. Therefore, while higher hardness may improve the wear resistance of the wheel, it must be carefully balanced against the wear behavior of the rail to optimize the overall system performance and cost-effectiveness.

**Figure 7:**
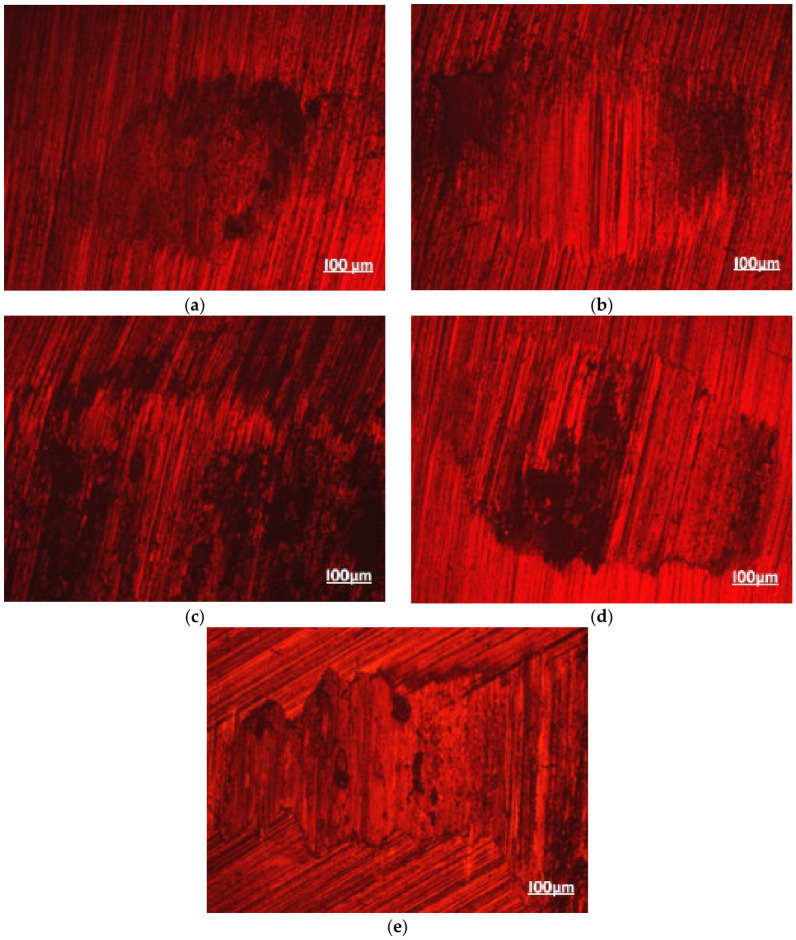
Surface of samples after the wear test: (**a**) 505 PTA, (**b**) 15 E, (**c**) initial material, (**d**) 36 C, and (**e**) 61 PTA.

Figure 7 presents micrographs of the abrasion surfaces resulting from the resistance-to-abrasive wear tests conducted on the samples. These micrographs provide detailed visual evidence of the damage induced by the forces applied during the tests. When the images are taken, the extent and nature of the surface degradation can be assessed under varying test conditions, including differences in the applied load, the test duration, and the pressure exerted on the sample surface. The micrographs reveal distinct patterns of wear damage in the different samples. Observations include the characterization of abrasion morphology, such as the presence of parallel grooves and scratches formed as a result of abrasive action, as well as cases where the entire surface layer was substantially worn away. Furthermore, the images allow for an evaluation of the retention and distribution of hard carbide particles embedded within the overlay material, which play a critical role in enhancing wear resistance. These insights are essential to understand the comparative performance of different coatings and the effectiveness of alloying treatments in improving the durability of surface layers under abrasive conditions.

In Figure 7a, at 400× magnification, a characteristic “scaled” structure is visible. This may indicate a microscale delamination process: cyclic block pressure causes the fatigue of the very thin surface layer, which begins to flake off, forming tiny wear flakes. The interiors of the grooves are not perfectly smooth. They contain transverse undulations, suggesting that the cutting process was not continuous but intermittent.

In Figure 7b, there are visible parallel grooves, and vertical scratches are clearly visible on the surface. This indicates microcutting by harder particles (like carbide fragments) or the roughness of the counter-sample. The edges of the grooves appear to pile up slightly, which is typical of a ductile metal matrix in which hard phases are embedded. Darker, irregular spots are visible in the central part of the image. The material exhibits the typical characteristics of a hard phase–matrix composite. The matrix wears faster, exposing the carbides, which can crack and fall out over time, leaving visible gaps (dark spots) in the image.

In Figure 7c, deep parallel scratches (grooves) are present on the surface, running vertically through the center of the wear scar. The image clearly delineates the boundary between the working area (a light, smooth strip with grooves) and the area without friction (a darker background on the sides), allowing for a precise assessment of the width of the wear scar. This material is based on an iron matrix, which, with a hardness of 60 HRC, offers significant resistance. However, the visible scratches are quite wide, which may suggest that the counter-sample material was equally hard. Compared to Durmat 15 E, the surface of Durmat 505 PTA appears to have more uniform and “cleaner” grooves, which may be due to differences in microstructure (e.g., different size or type of carbides in the matrix). Durmat 15 E (often Ni-based) typically exhibits a greater tendency toward plastic deformation of the matrix than harder Fe-based alloys such as 505 PTA.

In Figure 7d, taken at a higher magnification (×100), details of the failure mechanism can be seen due to the lower hardness; this material is highly susceptible to deformation. The visible grooves are wider and have more “blurred” edges compared to harder samples. The entire work surface is covered with a dense network of parallel scratches. At this magnification, it is clear that the hard particles penetrate deeply into the material structure, cutting microchannels. In the case of alloys with lower hardness levels, these particles can penetrate more easily back into the surface. Compared to Durmat 505 PTA, Durmat 36 C appears more “tired” and uneven. The higher hardness levels of the 505 PTA material allowed it to maintain surface continuity despite deep grooves, while Durmat 36 C shows more signs of structural deterioration and adhesion.

Figure 7e shows significantly greater magnification, allowing for the identification of the microstructural effects of friction. Compared to previous materials, there are dark, irregular “islands”. Also, cracks are visible that run perpendicular or diagonal to the direction of the crack. The material hardens under cyclic loading, then cracks and separates into sheets. Under the layer of dark deposits, fine parallel abrasion scratches are still visible, but they are much more subtle than those in the hard Durmat materials. The characteristic “smearing” of the surface suggests that the material was subjected to significant heating at the contact point, leading to its softening and plastic flow.

**Figure 8:**
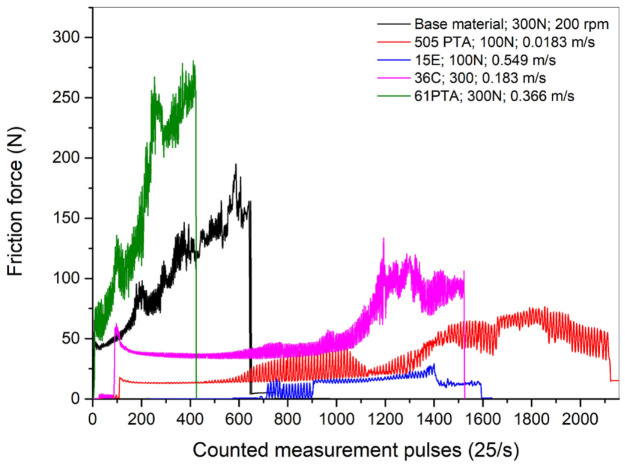
Results of the friction force test of the base material, 15 E material, 61 PTA material, 505 PTA material, and 36 C material.

**Figure 9:**
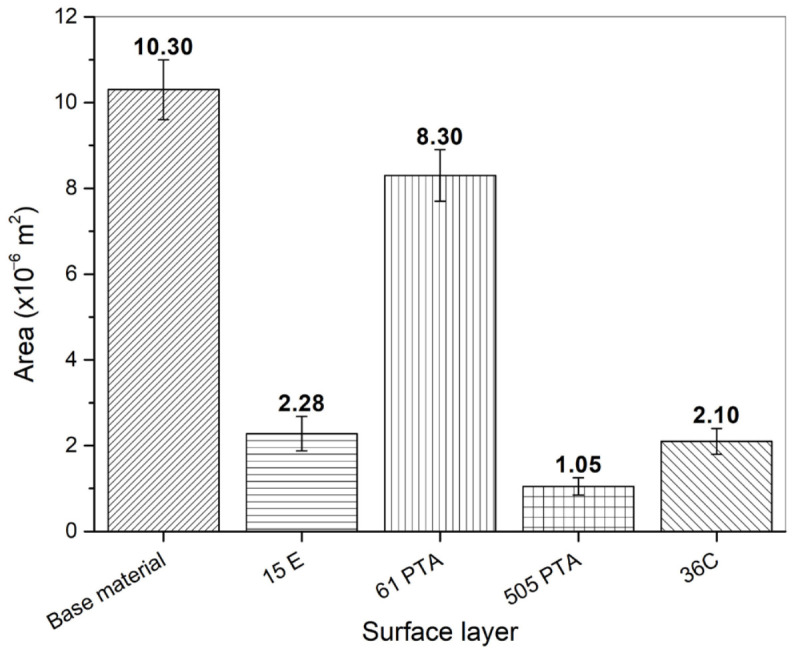
Wear test results of the wear area of the tribologically tested material.

In Figure 8, the friction force test result is presented, and in Table 3 the friction coefficients of the used powder material are detailed, whereas in Figure 9 the wear test results are shown.

It is worth paying particular attention to materials 36 C and 15 E, which, regardless of the test conditions, demonstrated virtually constant friction force, as well as low wear rates compared to other materials.

It can be seen that under similar load conditions of 300 N and a rotational speed of 200 rpm, the materials 15 E, 505 PTA, and 36 C maintain similar friction force. This suggests that under the present test conditions, the friction force fluctuations remain relatively stable.

Materials 36 C and 15 E demonstrate high friction force stability, similar to that of the parent material. The force curve in most tests of these materials is linear most of the time. The advantage of materials 36 C and 15 E is that they do not seize in a manner similar to that of material 505 PTA, which exhibited repeated impulse seizing. It would be worthwhile to undertake broader efforts to investigate the potential use of 36 C or 15 E materials in the regeneration and improvement of wheel flange properties. Research conducted and described in this paper supports the claim that materials 36 C and 15 E can, to some extent, improve the properties of railway wheels and extend their useful life. However, more research and experiments are necessary to determine the precise impact of the surfacing process on the durability of the wheels, as well as full-scale abrasive wear studies of wheels coated with these materials.

In Figure 9, the results of the wear test are presented. In particular, they indicate that the wear area systematically varies depending on the material properties: the material characterized by the highest hardness exhibits the smallest wear area, confirming its superior wear resistance, whereas the material with the lowest hardness shows the largest wear area, indicating the weakest wear resistance. The highest wear resistance area occurs in the case of the 505 PTA sample, with 1.05 × 10^−6^ m, which is ca ten times better than the wear resistance of the base material, whereas the base material and the 61 PTA material have the lowest wear resistance. Sample selection was determined using Hartley’s plan, so not all samples are analyzed. The goal of the study was not to compare all samples, but rather to identify the optimal one.

The surface discontinuities that occurred on the surface after PTA treatment can be attributed to complex interactions during the alloying process, such as gas entrapment, rapid solidification, or differences in thermal expansion between the base material and the alloying constituents. The observed differences highlight the influence of the alloying composition and processing parameters on the quality and integrity of the surface layers produced by PTA. Understanding these microstructural variations is critical for optimizing the PTA alloying process to enhance the wear resistance and overall performance of treated components.

Variations in the chemical composition, melting point, and thermal conductivity of the different alloying materials influence the heat transfer dynamics during the remelting process, thereby affecting the extent of the molten pool and the resulting surface morphology. Consequently, the choice of alloying materials plays a critical role in controlling the microstructural characteristics and surface quality of the remelted layers, which are key factors in determining the wear resistance and mechanical performance of the treated components. These findings underscore the importance of carefully selecting alloying materials and optimizing the processing parameters to achieve the desired surface characteristics. Additionally, the obtained results are consistent with the latest research [35] on the use of PTA technology in the regeneration and surface modification of machine components operating under conditions of intense wear. Passos et al. demonstrated that PTA-deposited coatings based on Fe–Cr–C–V alloys are characterized by high microstructural homogeneity and significant resistance to abrasive wear. The authors emphasized that a key parameter influencing the operational properties of the layers is the degree of dilution of the substrate material, which directly determines hardness and resistance to tribological degradation.

These results confirm that PTA technology is an effective tool in the regeneration of machine parts, enabling the production of layers with increased operational durability while simultaneously limiting the thermal impact on the base material. Consequently, this method can be successfully used not only in the regeneration of general-purpose components but also in the refurbishment of components exposed to intense wear, such as rolling elements or drivetrain components in rail vehicles.

PTA (Plasma Transferred Arc) technology opens up new possibilities for railway wheel remanufacturing in Europe, primarily because it combines high-quality metallurgical weld deposits with precise control of the thermal energy of the process, which is crucial under strict European safety standards (e.g., requirements for the structural integrity of the wheel rim, fatigue resistance, and allowable material defects). Unlike many remanufacturing methods used in other regions of the world, which allow for greater tolerance of structural discontinuities and quality deviations, PTA enables the production of layers with very low porosity, a lack of cracks, and a strong metallurgical bond to the substrate, which directly meets European operational requirements for wheel–rail system components.

An additional advantage of PTA is its limited heat-affected zone and ability to precisely control the degree of dilution of the substrate material, which allows for the preservation of the original mechanical properties of the wheel rim while restoring a working layer with high hardness and wear resistance. In practice, this means extending the service life of wheels without requiring complete replacement. This is particularly important in the European approach to rolling stock maintenance, where the priority is not only cost but, above all, predictable material degradation and compliance with rigorous certification procedures.

In this context, PTA can be a technological response to practices used in some regions of the world, where rail wheel remanufacturing is often performed using less advanced control methods (e.g., classic arc welding with greater dilution and greater quality variability). The European approach, however, requires technologies that minimize the risk of structural inhomogeneity and ensure process repeatability—and this is precisely where PTA offers a clear advantage, enabling the implementation of remanufacturing as a safe and certifiable alternative to replacing worn components.

## 4. Conclusions

In summary, the investigation demonstrated that the carbon steel substrate subjected to alloying through Plasma Transferred Arc (PTA) cladding with DURMAT 505 PTA BD-3.61 PTA and NiBSiWC powders successfully produced high-quality surface layers. These layers were characterized by a generally defect-free microstructure that exhibited an absence of cracks and other common surface imperfections. The steel structure of the wheel remains essentially unchanged after thermal spraying, and any thermal impact is only superficial, as revealed by TEM investigations. Therefore, this process does not significantly affect the rim’s mechanical properties or operational durability.

Among the materials tested, the 61 PTA alloy was found to be unsuitable for practical application in the context of railway wheel refurbishment or enhancement. This unsuitability is mainly due to the high risk of defects observed in the alloyed layers, such as porosity and cracking, which compromise structural integrity.

Untreated native carbon steel exhibited the greatest variability in friction force under the range of test conditions. This variability suggests that the wear rate of the untreated material is highly sensitive to operational parameters such as load, speed, and contact conditions. In particular, under low-speed but high-load conditions, the untreated steel showed a comparatively lower wear rate than the coated materials. In contrast, at elevated rotational speeds, the untreated material experienced dramatically increased wear, with rates approaching nearly eight times those of the alloyed coatings. This finding underscores the critical advantage of applying surface treatments to enhance performance in demanding high-speed operating conditions.

Although the 61 PTA and 505 PTA alloys may not be optimal for improving the mechanical properties of the railway wheels themselves, these materials possess potential utility regarding the wear resistance reaching even ca 10% of the value of the base material’s wear for the 505 PTA material and over 20% for the 15 E powder. Specifically, its application could be highly beneficial for the restoration of friction plates in axle boxes and various hydraulic systems. Furthermore, the treated layers showed a significant increase in hardness relative to the untreated base material, reaching even 248% for the PTA505 material, which is a critical factor in improving the resistance to wear and extending the service life of the components.

## Figures and Tables

**Table 1:** Chemical compositions (wt.%) of the steels studied.

Steel Type	C	Si	Mn	P	S	Cr	Cu	Mo	Ni	V	Cr+Mo+Ni
ER7	0.52	0.40	0.80	0.020	0.015	0.30	0.30	0.08	0.30	0.06	0.50

**Table 2:** Chemical compositions (wt.%) of the PTA powders.

Powder Type 61 PTA	C %	Si %	Ni %	B %	Fe %	Hardness, HRC	Melting Point, °C	Density, g/cm^3^	Grain Size, µm
	<0.1	3.0	Base	3.0	<2.0	70	1070	8.1	160
505 PTA	C %	Cr %	Mo %	Fe %	-				
	2.4–2.8	<7	1.00–1.25	Base	-	79	1310	8.0	160
15 E	Ni %	Cr %	Fe %	Si %	C %				
	Base	17	4	4	1	63	1024		106
36 C	Cr %	Fe %	Si %	C %	Ni %				
	11	2.5	2.5	0.5	Base	70	1025	8.3	150

**Table 3:** Friction coefficients of the tested samples.

Sample, Material	Maximum Value of the Friction Coefficient	Maximum Friction Force (N)
505 PTA, 100 N	0.77	76.5
14 E, 100 N	0.47	235.8
36 C, 300 N	0.45	133.6
Base material	0.65	195.1
61 PTA, 300 N	0.94	280.7

## Data Availability

The original contributions presented in this study are included in the article. Further inquiries can be directed to the corresponding author.
